# Computer modeling reveals modalities to actuate mutable, active matter

**DOI:** 10.1038/s41467-022-30445-x

**Published:** 2022-05-16

**Authors:** Abhrajit Laskar, Raj Kumar Manna, Oleg E. Shklyaev, Anna C. Balazs

**Affiliations:** Department of Chemical Engineering, University of Pittsburgh, Pittsburgh, PA 15260 USA

**Keywords:** Nanoscale devices, Design, synthesis and processing

## Abstract

Catalytic reactions on flexible sheets generate fluid flows that transform the shape of the sheet, which in turn modifies the flow. These complex interactions make computer models vital for designing and harnessing these feedback loops to create soft active matter that autonomously performs self-sustained mechanical work.

## Forms of soft active matter

**Fig. 1:**
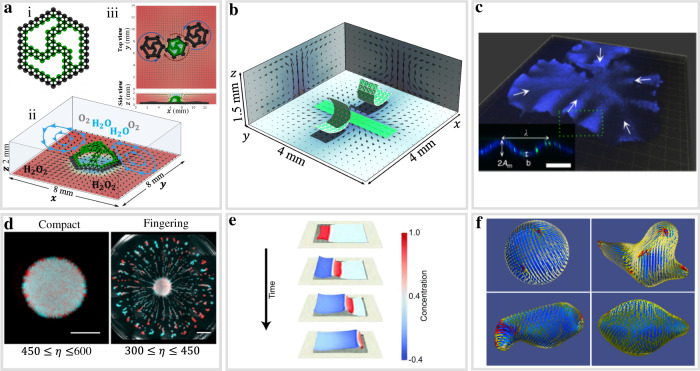
Active, flexible sheets actuating and assembling soft active matter. **a** Initially flat sheet is immersed in a fluid-filled chamber. Active, catalase-coated (green) parts of the sheet generate a fluid flow (blue arrows in ii) that morphs the initially flat shape (i) into a three-dimensional structure (ii). The asymmetry of the structure enables the active rotor (ii), to rotate in the clockwise direction. The side view (the bottom of panel iii) shows that lighter, central regions of the sheet pop up due to the generated flow. Cogs attached to the side of the active gear (iii) enable the rotation of two passive wheels (in black). **b** Two catalase-coated sheets in the presence of an immobile catalytic patch (green rectangle) form coupled oscillators, which dynamically assemble into structures that become synchronized in both space and time. **c** Spontaneous buckling of acto-myosin sheet. Inset shows the wavelength, final gel thickness, and buckling amplitude. The scale bar is 100 $\mu m$. **d** Morphologies of a yeast colony on a viscous substrate with viscosity $\eta =600\pm 90{Pa\; s}$ after 72 h of growth (left panel), and with viscosity $\eta =450\pm 70{Pa\; s}$ after 84 h of growth (right panel). The scale bars in left and right panels correspond to 5 and 10 mm. **e** Self-propulsion of deformable elastic sheets driven by chemical waves propagating through the sheets. **f** Deformations of active shells generated by self-induced flows of active particles. The spherical shell (top left) is deformed by extensile (top right) and contractile forces (bottom panels) generated in the system. Panel **a** is adapted from ref. ^9^, Panel **b** is adapted from ref. ^13^. Panel **c** is adapted from ref. ^14^. Panel **d** is adapted from ref. ^15^. Panel **e** is adapted from ref. ^7^. Panel **f** is adapted from ref. ^6^.

Chemically active matter commonly refers to nano- and micro-scale objects in a solution that consumes energy from catalytic reactions in the fluid to perform mechanical work. In converting reactants into products, these catalytic reactions generate local gradients in the concentration of chemicals and density of the fluid. The gradients propel the fluid to move and enable the system to mimic salient features of chemo-mechanical transduction in living organisms, which convert energy from nutrients into mechanical action (e.g., metabolism and motion). If the catalysts driving this biomimetic behavior are anchored to an immobile surface in a fluid-filled microchamber, then the system acts as a chemical “pump”, which drives the fluid to flow; the flow, however, does not alter the location of the immobilized catalysts. If the catalytic reaction occurs on hard, mobile particles (e.g., spheres or rods), the chemically driven flow drives the particles’ motion, creating chemical “motors”, but does not deform the hard active matter. The situation is significantly more complicated if the catalysts are attached to soft active matter (SofAM), such as movable, flexible sheets. Now, the hydrodynamic interactions not only drive the sheets’ motion but also morph this elastic layer into three-dimensional shapes (Fig. 1a) or drive new modes of self-organization (Fig. 1b). As discussed below, one challenge for taking full advantage of SofAM is developing predictive models that capture the dynamic interplay among the material’s elastic properties, hydrodynamic interactions, and the kinetics of the chemical reactions. With robust models, researchers could uncover new approaches to actuate biomimetic soft matter and create chemically driven, flexible machines with exceptional levels of autonomous, self-sustained functionality^1^. Rather than requiring external electrical or mechanical power, the “nutrients” for operating SofAM can simply be carried in a small vial of chemicals.

Since the discovery^2^ in 2014 that enzymatic reactions on immobile surfaces generate fluid flow, researchers have examined catalyst-coated, mobile surfaces, such as zero-dimensional (0D) rigid particles and one-dimensional (1D) flexible filaments^3^. To date, however, there have been few experiments on 2D or 3D soft active matter, which introduces additional degrees of freedom and complex dynamics. For example, computational models reveal that a soft catalyst-coated 2D layer can repeatedly bend and unbend to mimic the locomotion of inch worms^3^. Such distinctive dynamic behavior is due to a feedback loop (Fig. 1a) coupling the chemically triggered hydrodynamic forces that act on the sheet, and the force exerted by the sheet on the surrounding fluid (fluid–structure interactions).

Due to their flexibility, SofAM can exhibit the mutability of living systems. For instance, one sheet coated with different catalytic patches can be reconfigured into multiple shapes, depending on the reactant added to the solution, and thus, allow one sample to perform a range of distinct functions^3^. One challenge in effectively utilizing SofAM is establishing routes to harness the inherent feedback loops in the system (Fig. 1a). Namely, researchers must co-design the SofAM and the chemically generated flows so that the dynamic crosstalk between these components yields the desired structural and temporal evolution. These essential fluid–structure interactions, however, depend on a large number of parameters, including the elasticity, size, geometry, and the number of immersed sheets; arrangement of different catalytic patches on each sheet; types of catalytic reactions and reaction networks (e.g., cascades of coupled reactions); dimensions of the chamber, and presence of catalytic patches on the chamber walls. Moreover, the systems not only dissipate energy but also encompass interconversion across different energy domains (e.g., chemical, hydrodynamic, mechanical domains, optical and thermal). With this level of complexity, experimental approaches would necessitate expending considerable synthetic effort and performing numerous investigations to probe the multi-dimensional phase space. Given the time and expense of methodically analyzing the influence of all the different variables, the experimental studies could potentially miss key interactions and limit our understanding of SofAM. Conversely, multi-physics computer models allow researchers to perform systematic studies, which are necessary to describe the chemo-hydro-mechanical behavior of a single entity and uncover new forms of self-assembly among multiple entities. Such systematic studies are vital for the development and effective utilization of SofAM.

Multi-physics simulations have only recently been developed for SofAM^4–7^. While these models capture salient features of mobile, elastic surfaces, they, however, neglect either the host fluid^7,8^; reactions occurring in the solution^4^, or the specific chemistry involved in the reactions^6^. Building on the latter studies, our group^3^ developed a computational approach to solve the coupled equations for (1) flow in a fluid-filled chamber; (2) advection and diffusion of chemicals in the solution and their reactions with deformable materials; (3) dynamic behavior of the nodes that make up the elastic material; and (4) fluid–structure interactions between these nodes and the solution (see Fig. 1a). Notably, such coupled interactions are resplendent in biomaterials. For example, all these steps are present as actin-myosin sheets undergo enzymatic reactions in a solution that cause the contraction of the elastic layer and generation of local fluid flow; the resulting fluid–structure interactions drive the sheets to buckle, as shown in Fig. 1c. In Fig. 1d, metabolic activities in thin films of yeast cells lead to density gradients that propel the surrounding fluid, which breaks the initial film into smaller units and thereby helps the dispersion and propagation of the colony.

Below, we illustrate how computational approaches capturing the above (1)-(4) interconnected processes can facilitate the design of synthetic SofAM that perform chemically driven, mechanical work, and thus serve as chemical gears or self-oscillating chemical clocks. More generally, the results reveal new modes of actuating and controlling the transformation of 2D biomimetic soft matter into 3D functional materials.

## Actuating active sheets

Figure 1a shows a deformable, catalyst-coated sheet that resembles a flat wheel, with spokes that interconnect an outer rim and inner center^9^. The center of the sheet is pinned to restrict its horizontal motion but is free to move in the vertical direction. The entire sheet is coated with the enzyme catalase, with the nodes in black being heavier than the ones in green. The addition of hydrogen peroxide activates the catalyst to decompose H_2_O_2_ into the lighter products, water and oxygen, and thereby generate buoyancy-driven fluid flow. The generated flow drives the lighter, central region of the sheet to pop up out of the plane, morphing the 2D layer into a 3D shape. The sheet, in turn, exerts forces on the fluid that modify the flow; in particular, the distended spokes act as blades that “catch” the flow and rotate the 3D object. The computer models allowed us to capture and visualize the latter feedback between the fluid and sheet and design the optimal sheet architecture to maximize this fluid–structure interaction. The computer models thereby reveal new routes for actuating flexible material and achieving functionality that is not possible with chemically active matter that is immobile or mobile and inflexible.

With the addition of cogs to the outer rim, the active structure (in green in Fig. 1a(iii)) operates as gear, which activates the motion of passive (non-coated) units (in black). Namely, the green cogs exert a mechanical force on the neighboring cogwheels and thus rotate the passive units in a controllable direction. These results provide a design rule for creating simple machines that undergo self-sustained motion within fluidic devices.

In addition to chemically generated fluid flows, alternative modeling approaches have revealed other forces that can drive 2D elastic sheets to undergo distinctive locomotion. In particular, chemical waves traveling through a flat sheet can generate non-uniform stresses, which drive the sheet to undergo peristaltic-like motion as it effectively crawls on a dry frictional surface (Fig. 1d). Since the 2D sheet can conform to a geometrically patterned substrate (in contrast to a 1D object), the sheet’s movement can be tailored by varying the underlying surface topology.

Beyond the dimensionality of flat sheets, researchers have recently modeled active deformable shells^6,8,10^; to gain insight into the dynamic behavior of biological membranes^11,12^. Figure 1e shows deformations of an initially spherical, elastic shell composed of active nematic particles. The reorientation of these elongated particles introduces topological defects on the shell’s surface and generates the flow of the surrounding fluid. The synergistic interactions among the elastic properties of the shell, the dynamic behavior of the topological defects, and the flowing fluid drive the self-morphing of the shell into distinct non-spherical shapes (Fig. 1e). These results shed light on the morphogenesis of biological cells and tissues and can guide the design of bioinspired soft materials.

## Driving the self-organization of self-oscillating SofAM

The synchronization of self-oscillating systems is vital to various biological functions, from the coordinated contraction of heart muscle to the self-organization of slime molds. Through modeling, Balazs and co-authors designed bioinspired materials systems that spontaneously form shape-changing, self-oscillators, which communicate to synchronize both their temporal and spatial behavior^13^. The approach utilizes both immobile and mobile surfaces in the microchamber. The green regions in Fig. 1b show that a portion of the bottom, stationary wall, and two movable sheets, which initially lie on either side of this patch, are coated with catalase. With the addition of H_2_O_2_, the active, immobile patch and the sheets generate an inward fluid flow (marked in black) centered at the patch. The combined hydrodynamic forces pull the sheets toward this center, while the excluded volume interactions keep the sheets apart. Through the inherently coupled interactions, the fluid flow affects the shape and movement of the flexible sheets, which in turn modify the fluid flow. This distinctive feedback, which combines hydrodynamic, steric, and fluid–structure interactions, causes the sheets to form self-regulating coupled oscillators, whose motion is synchronized in time and space. The phase dynamics of the coupled self-oscillators can be tuned by altering the extent of catalyst coverage on either the sheets and/or the surface patch. This breadth of dynamic behavior expands the functionality of the coupled oscillators, enabling soft robots to display a variety of self-sustained, self-regulating moves.

## Future challenges

Creating predictive models for controlling the actuation and motion of SofAM will require further developments in theory and simulation. While the above discussion focused on buoyancy-driven flow, two or more mechanisms for propelling fluids (such as diffusiophoresis, electrophoresis, and Marangoni effects) can be triggered simultaneously by a single chemical stimulus. Models that capture these simultaneous processes can reveal new means to control the surface and bulk flows independently and thus, provide effective approaches to separately direct the self-organization of the immersed sheets and particles or autonomously sort different sized particles in microchannels^3^.

As described above, the spatiotemporal behavior of SofAM sheets typically involves multiple coordinated, dynamic processes; capturing these processes within computationally feasible timescales introduces new challenges for modeling these systems in 3D. For example, the model of the spontaneous buckling of an acto-myosin sheet (Fig. 1c) encompasses the coordinated contraction of the sheet’s edges, the autonomous generation of density gradients driven by these contractions, and the ensuing fluid flow^14^. Modeling this integrated behavior in SofAM to understand and ultimately direct their dynamic assembly could require simulations involving hundreds (or thousands) of actin filaments, myosin motors and cells. More coarse-grained modeling approaches are needed to simulate systems of that size scale in reasonable run times.

The above studies focused on the conversion of chemicals into mechanical energy. Chemical reactions driven by light (or other stimuli) can involve multiple energy domains, and thus require models for photo-chemo-thermo-mechanical transduction. To capture this coupling of energy fields also requires longer simulation run times, as well as accurate models that describe the interaction among all these dynamic processes.

Another challenge for modeling is to simulate cascade reactions that provide a programmable delay between individual chemical reactions. This delay is critical for directing particle motion in multi-step processes. For example, with regulation, a reaction yielding dense products can lead to outward motion (away from a catalytic patch), before the next reaction, which produces less dense products, would lead to inward motion. In other words, coordination between the chemical reactions will modulate directed transport (whereas simultaneous activation of both reactions would cancel out the fluid motion). Simulating cascades involving a number of reactions may require the development of new coarse-grained models, but meeting this goal would allow researchers to design multi-stage processes that perform autonomously in microchambers.

All these challenges provide guidelines for new research directions, which can inspire the imagination of scientists for a considerable time.
